# Effects of Litterfall on the Accumulation of Extracted Soil Humic Substances in Subalpine Forests

**DOI:** 10.3389/fpls.2020.00254

**Published:** 2020-03-05

**Authors:** Xinyu Wei, Yulian Yang, Ya Shen, Zihao Chen, Yuliang Dong, Fuzhong Wu, Li Zhang

**Affiliations:** ^1^Key Laboratory of Humid Subtropical Eco-geographical Process of Ministry of Education, Fujian Normal University, Fuzhou, China; ^2^Long-Term Research Station of Alpine Forest Ecosystems, Key Laboratory of Ecological Forestry Engineering, Institute of Ecology and Forestry, Sichuan Agricultural University, Chengdu, China; ^3^Ecological Security and Protection Key Laboratory of Sichuan Province, Mianyang Normal University, Mianyang, China

**Keywords:** litterfall, humic substances, humic acid, fulvic acid, soil organic matter, subalpine forest

## Abstract

Plant litter is one of the main sources of soil humus, but which can also promote primary humus degradation by increasing microbial activity due to the higher availability of energy released, resulting in a confusing relationship between litterfall and soil humus. Therefore, an *in situ* incubation experiment was carried out in three subalpine forests (coniferous, mixed and broadleaved forests) on the eastern Qinghai-Tibetan Plateau. We set up two treatments. One that allowed litterfall to enter the soil normally and the other prevented litterfall to enter the soil. Soils were sampled in October (the end of the growing season), January (the onset of the freezing season), March (the end of the freezing season), and May (the start of the growing season) from May 2017 to May 2018. By assessing the litterfall production, the content of total extracted humus, humic acid (HA) and fulvic acid (FA) in the topsoil (0–20 cm) in each incubation period, we determined the impact of litterfall on the content of humus extracted from the soil during the freezing and the growing season. Over 1-year incubation, soil total extracted humus and HA showed considerable decreases in the treatment of retained litterfall in the mixed forest but not in the coniferous or broadleaved forests. Moreover, litterfall significantly reduced the contents of soil total extracted humus and HA during the growing season in all three forests, while only reduced soil HA content in the broadleaved forest in the freezing season. The relationship between litterfall and soil extracted humic substances was greatly regulated by the seasonal dynamics of litter types and litter production in all forest types. The larger the amount of litterfall was, the more litterfall could promote the reduction of soil extracted humic substances. Compared with a single type of broadleaf or needle litter, mixed litterfall could promote a higher degradation of soil humic substances. However, broadleaf litter might lead to much greater decreases in soil humic substance than needle litter because it is more decomposable. These results indicate that the effect of litterfall on soil humic substances are mainly regulated by litter types and litter production. Moreover, the effects of litterfall on soil humic substances are more significant during the growing season than winter. This suggests that the longer growing season and a shorter winter caused by ongoing global warming may alter the relationships between litterfall and extracted humic substances, further disrupting the carbon balance of forest ecosystems in the subalpine forests.

## Introduction

Humus is the main component of soil organic matter (Kogel-Knabner, 2000) and is important for soil fertility and nutrient cycling (Ponge, 1999; Ono et al., 2011; Chertov and Komarov, 2013). As a basic carrier of nutrients and carbon, forest litterfall plays an essential role in the formation of soil humus (Stevenson, 1994; Prescott et al., 2000; Neumann et al., 2018). Different kinds of litterfall have different chemical compositions and qualities that might determine the development processes of humus in soil (Wei et al., 2018; Zanella et al., 2018a, b). However, there is evidence that shows that the incorporation of fresh organic matter into soil can increase microbial activity due to the higher availability of energy released, then exacerbate soil humus mineralization as named “priming effect” (Broadbent and Nakashima, 1974; Wu et al., 1993; Liljeroth et al., 1994). As such, it is still unclear whether the input of litterfall promotes the synthesis or degradation of soil humic substances. Moreover, both climatic factors (Prescott et al., 2000; Ponge, 2013) and forest litter type (Langenbruch et al., 2012) significantly affect litter humification and soil humus development, which further complicates the relationship between litterfall and soil humic substances.

Litterfall production often varies seasonally (Ma and Wang, 1993; Yang et al., 2017). Different litterfall production has different effects on soil humic substance content. Increasing fresh litterfall inputs increases the amount of CO_2_ released from the soil, indicating that there is a positive correlation between the decomposition of soil organic matter and the amount of litterfall (Jenkinson, 1977; Leff et al., 2012; Xu et al., 2013). Many studies have also shown that the relationship between soil organic matter and nutrient availability is related to the litter quality (e.g., C/N ratio) (Kuzyakov, 2002; Cheng, 2009). When the C/N ratio of twig litter is high (Yang et al., 2007), microorganisms are more likely to mine N from soil organic matter and thus increase its decomposition (Kuzyakov, 2010). Studies also found that the relative proportions of different litterfall components change with time, resulting in the accumulation or decomposition of soil humic substances content (Fontaine et al., 2003; Kuzyakov, 2010). Litterfall is often dominated by foliar litter during the growing period and by twigs in winter. Foliar litter, which is rich in liable components and nutrients, is more likely to stimulate microbial activity and further promote soil humification and “old humus” degradation (Gonet and Debska, 1998). In contrast, the addition of twig litter, which is rich in refractory substances such as lignin, could stay more readily in soil as the components of soil humus than easily decomposable compounds (Fontaine et al., 2003; Kuzyakov, 2010). Therefore, the effects of litterfall on soil humus content may vary at different seasons. In addition, global warming not only increases litter production (Delucia, 1999) but also increases the litter decomposition rate (Chapin and Shaver, 1996; McHale et al., 1998), which complicates the relationship between litterfall and soil humic substances content.

Extracted humic substances are mainly divided into humic acid (HA) and fulvic acid (FA) (Abakumov et al., 2013; Kõlli and Rannik, 2018); these substances are of great significance to soil fertility, nutrient cycling, and the sustainability of ecosystem productivity (Komarov et al., 2016). FAs are a group of substances that are more active than HAs due to their small molecular weight, aromaticity, and stronger acidity (Wen, 1984). However, FA is easily lost through physical leaching by rainfall and snowmelt water (Elliott, 2013; Ni et al., 2015). High temperatures and year-round low temperatures will prevent the accumulation of soil HA (Wen, 1984). Studies have shown that climate, substrate quality, and the soil environment determine whether the formation of humic substances is dominated by HA or FA (Stevenson, 1994) due to their special properties of acid solubility and alkali solubility (Prescott et al., 2000; Ponge and Chevalier, 2006). However, there are still many unclear factors regarding the formation and transformation of humic substances.

As an important ecosystem in southwestern China (Yang et al., 2005), the subalpine forests in western Sichuan play an important role in the regional and national economy as well as in regulating the climate and conserving water and soil (Zhang et al., 2004; Sun et al., 2005; Wu et al., 2010). The accumulation of soil humic substances is essential for soil fertility and maintaining the stability of subalpine forest ecosystems, but it is closely related to litterfall. However, frequent freeze-thaw cycles and long-term low temperatures in winter can cause physical damage and chemical changes in litterfall further inhibiting the accumulation of humic substances by providing a fast-turn-over substrate for living microorganisms (Deng et al., 2010; Wetterstedt et al., 2010), and freeze-thaw cycles can even destroy the structure of newly formed humus (Dou, 2010) in subalpine forests. These processes could offset the contribution of litter to soil humus. Therefore, the effects of litter on the content of soil humus in subalpine forests remain uncertain.

We hypothesized that retained litter may decrease soil humic substances content, but this effect may be regulated by litter types and litter production in different periods. To test this hypothesis, we selected three representative forests (including coniferous, mixed and broadleaved forests) that dominate the subalpine forests on the eastern Tibetan Plateau, and check the effects of litter input on soil humic substances content as affected by seasonal freeze-thaw from May 2017 to May 2018. Our objective is to assess the accumulation or loss of soil humic substances with and without litter inputs.

## Materials and Methods

### Study Site

The study site was located in the Wanglang National Nature Reserve (103°55′−104°10′ E, 32°49′−33°02′ N, 2540–2600 m), Pingwu County, Sichuan Province, China. The mean annual temperature ranges from 2.5 to 2.9 °C, and the maximum and minimum temperatures are 26°C (July) and −18°C (January), respectively. The annual mean precipitation is approximately 826 mm, with most falling between May and August. The winter normally extends from late October to late April (Yang et al., 2004). The soil type at the experimental sites is dark brown forest soil according to the Chinese soil genetic classification (Gong et al., 2007) and is classified as a type of Cambisol in the world reference base for soil resources [FAO, 2006 (2007)].

The geological characteristics, dominant arboreal species and representative shrubs of the sites are shown in Table 1.

**TABLE 1:** Altitude, slope, dominant arboreal species and representative shrubs of the coniferous forest, mixed forest and broadleaved forest in the study site.

Forest type	Altitude(m)	Slope(°)	Dominant species	Major understory vegetation
Coniferous forest	2600	25	*Picea purpurea*	*Lonicera japonica*
				*Rubia cordifolia*
				*Adiantum capillus-veneris*
Mixed forest	2580	30	*Abies faxoniana*	*Rhododendron lapponicum*
			*Picea purpurea*	*Fargesia denudata*
			*Betula albosinensis*	*Artemisia lactiflora*
Broadleaved forest	2540	22	*Tilia tuan*	*Ribes nigrum*
			*Padus racemosa*	*Fargesia denudata*
			*Salix paraplesia*	*Elaeagnus pungens*
				*Rubia cordifolia*
				*Lonicera japonica*

### Experimental Design

**FIGURE 1:**
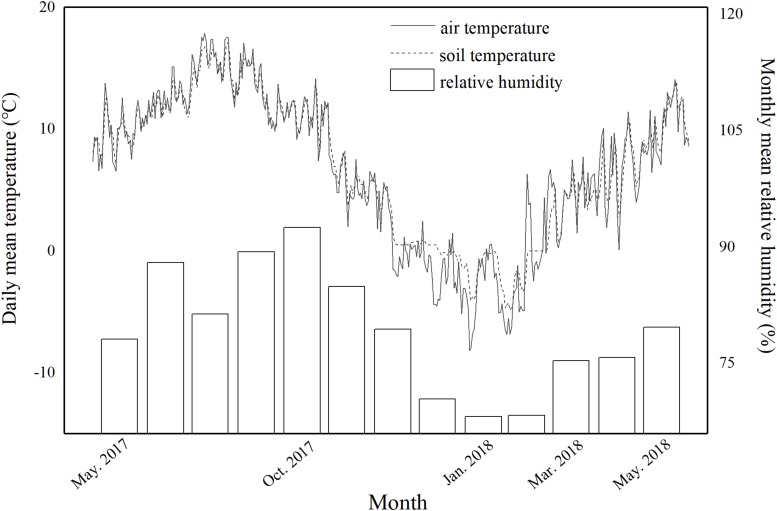
Daily mean temperature of the soil and air and the monthly mean relative humidity of the air in a subalpine forest on the eastern Tibetan Plateau from May 11, 2017 to May 30, 2018.

This study was conducted in coniferous, mixed and broadleaved forests with similar elevations and age structures (Table 1). Three plots were established in each forest type at similar altitude, slope and aspect. In May 2017, we set twelve *in situ* incubation boxes with lengths of 70 cm, widths of 51 cm and heights of 43 cm in each plot, a total of 108 boxes (12 × 3 plots × 3 sites = 108). We dug to about 50 cm depth, removed litterfall, stones, impurities, etc., from the soil and then added the soil to the corresponding *in situ* incubation boxes. Small holes were drilled in the bottoms of the boxes to ensure that water flow could permeate without removing soil. We set eleven boxes as the litterfall removal boxes and one box as the permanent litterfall input box in each plot. For the permanent litterfall input boxes, no interception net was set above the box. This allowed continuous accumulation of natural input of litter. The litterfall removal boxes meant that the boxes did not accept input of litter. In the litterfall removal boxes, nylon net was fixed 50 cm above the soil surface with brackets. This net captured all falling litterfall and prevented it from reaching the soil. We collected all the litterfall on the nylon net on each sampling date. The daily mean air temperature, air humidity and soil temperature were measured to quantitatively assess the environmental factors. The air temperature and humidity at the three study sites were measured using air temperature and humidity recorders (LITE5032P-RH, Fourtec-Fourier Technologies, Israel) and shown in Figure 1. Button thermometers (iButton DS1923-F5, Maxim/Dallas Semiconductor, Sunnyvale, United States) were randomly buried in the boxes at the three study sites to record the soil temperature.

Based on temperature detection data and previous studies conducted by our group (Wu et al., 2010), the period between two adjacent sampling dates were named the growing season (May 11, 2017–October 29, 2017), early freezing season (October 29, 2017–January 16, 2018), deep freezing season (January 16, 2018–March 24, 2018), and early growing season (March 24, 2018–May 30, 2018).

### Sample Collection and Treatment

**FIGURE 2:**
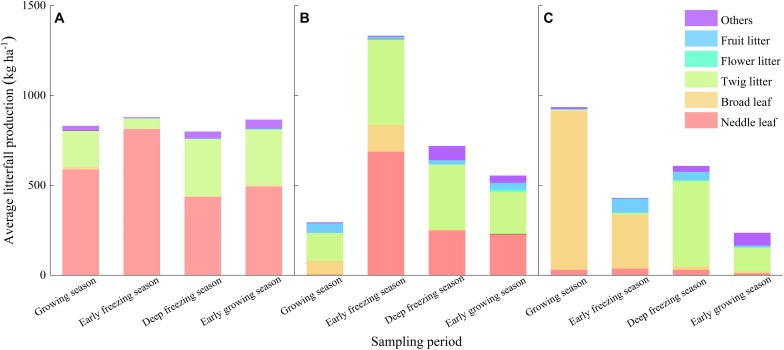
Average production of different litter components in the three forests in the different sampling periods on the eastern Tibetan Plateau: **(A)** coniferous forest, **(B)** mixed forest, and **(C)** broadleaved forest.

From May 2017 to May 2018, samples of soil and litterfall were collected at the end of the growing season (October), the onset of the freezing season (January), the end of the freezing season (March), and the start of the growing season (May). First, all the litterfall on the nylon net of the litter removal boxes was collected on each sampling date. All the litterfall from the same plot was evenly mixed and brought back to laboratory to calculate the average litterfall production (Figure 2). Second, the nylon net was removed from one randomly selected litterfall removal box in each plot on each sampling date to allow that box to re-receive litterfall from that time onward. Third, separate soil samples were collected from all permanent litterfall input boxes in every plot on each sampling date. Finally, we collected soil samples from the boxes that were just removed from the nylon net on the sampling date. When collecting the soil samples, the upper litterfall was removed, and the topsoil (0–20 cm, the average depth of soil organic layer in the forest) was collected randomly and mixed uniformity in each *in situ* incubation box. All litter materials and soil samples were air-dried at room temperature for 1 week. Then, the litter materials were classified as needle leaf, broad leaf, twig litter, flower or fruit litter, and any unrecognized residue was classified as other (including unidentifiable plant residues and animal waste). All soil samples were ground and passed through a 0.25 mm sieve in preparation for the extraction of humic substances.

### Sample Analysis

The extraction and separation methods of soil extractable total humus, HA and FA were as follows. The air-dried samples (0.500 g) were shaken with a mixed solution of 100 mL of 0.1 mol L^–1^ NaOH+0.1 mol L^–1^ Na_4_P_2_O_7_ for 10 min and heated at 100^°^C in boiling water for 1 h (Adani and Ricca, 2004; Wang et al., 2010). The extracted liquid was filtered, and the filtrate was used to analyze the soil extractable total humus. Simultaneously, 20 mL of extracted liquid was collected, and 0.5 mol L^–1^ H_2_SO_4_ was used to separate the HA and FA fractions at 80°C. Then, the HA was dissolved with a hot 0.05 mol L^–1^ NaOH solution (Kumada et al., 1967). The humus and HA fractions were passed through a 0.45 μm filter and then analyzed for the concentrations of humus and HA using a total organic carbon (TOC) analyzer (vario TOC cube/vario TOC select, Elementar Analysensysteme GmbH, Hanau, Germany).

### Calculations and Statistical Analysis

The concentrations of humus and HA were analyzed, and the concentration of FA was calculated as follows (Gigliotti et al., 1999):

(1) C(gkg)-1F⁢A=C-h⁢u⁢m⁢u⁢sCH⁢A

where C_*humus*_, C_*HA*_ and C_*FA*_ are the contents of total extracted humus, HA, and FA, respectively.

The HA/FA ratios were calculated from the current measured HA and FA contents on each sampling date (Abakumov et al., 2013).

The daily average temperature, number of freeze-thaw cycles were calculated for the different sampling periods based on the temperature data. Freeze-thaw cycles were defines as periods where the temperature was above or below freezing for 3 h until it changed to below or above freezing again (Konestabo et al., 2007).

An analysis of variance (ANOVA) was used to test for significant (*P* < 0.05) differences in the contents of soil total extracted humus, HA and FA and the HA/FA ratios between the litter retained and removed plots at each period. Correlations between the contents of total extracted humus, HA and FA and the HA/FA ratios and environmental factors under different litter treatments were analyzed by Pearson correlation analysis. The above analyses were performed using SPSS 20.0 (IBM SPSS Statistics Inc., Chicago, IL, United States), and the figures were drawn with Origin Pro9.0 (OriginLab, Northampton, MA, United States).

## Results

### Total Extracted Humus

**FIGURE 3:**
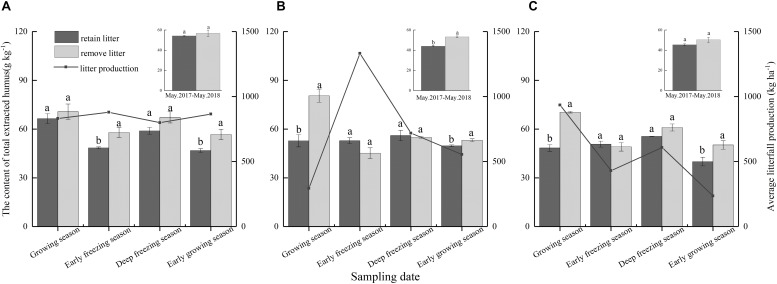
Content of soil total extracted humus was affected by litter removal and average litterfall production at different periods in the **(A)** coniferous forest, **(B)** mixed forest, and **(C)** broadleaved forest. The histograms show the averaged values over 1 year. The lines show the averaged litterfall production in each forest. The presented content values are the means of 3 observations; the error bars represent standard errors. Lowercase letters represent significant differences in the contents of total extracted humus between the different litterfall treatments.

The litterfall input of subalpine forests significantly affected the content of soil total extracted humus (*F* = 27.35, *P* < 0.01, Table 2), and there were significant differences among forest types and incubation periods (Table 2). Over the 1-year incubation, litterfall significantly decreased the soil total extracted humus content in the mixed forest (Figure 3B), while there were non-significant effects in the coniferous and broadleaved forests (Figures 3A,C). Continuous litterfall input reduced the soil total extracted humus content by 17.5% in the mixed forest. However, litterfall significantly decreased the content of soil total extracted humus in all three forests during the early growing season and the growing season rather than in the other periods (Figure 3). The inhibitory effects of litterfall on the soil total extracted humus in the early growing season was 20.4% for broadleaved forest, 17.5% for coniferous forest, and 6.5% for mixed forest. In contrast, litterfall reduced the soil total extracted humus by 34.5, 31.2 and 6.1% in the growing season in the mixed forest, broadleaved forest, and coniferous forest, respectively.

**TABLE 2:** Repeated measures ANOVA results for the effects of incubation period, forest type, litter, and their interactions on soil total extracted humus, humic acid, and fulvic acid.

Factors	df	Total extracted humus		HA		FA	
		F	sig.	F	sig.	F	sig.
Period	3	68.36	0.00**	37.80	0.00**	16.02	0.00**
Type	2	5.82	0.02*	11.79	0.00**	2.24	0.15
Litter	1	27.35	0.00**	25.25	0.00**	11.36	0.01*
Period × Litter	2	19.04	0.00**	12.41	0.00**	8.52	0.00**
Period × Type	6	3.00	0.04*	6.54	0.00**	3.27	0.03*
Type × Litter	3	0.48	0.63	1.60	0.24	1.12	0.36
Period × Type × Litter	6	8.99	0.00**	12.60	0.00**	5.65	0.00**

*P < 0.05, **P < 0.01. *n* = 36.

### Humic Acid

**FIGURE 4:**
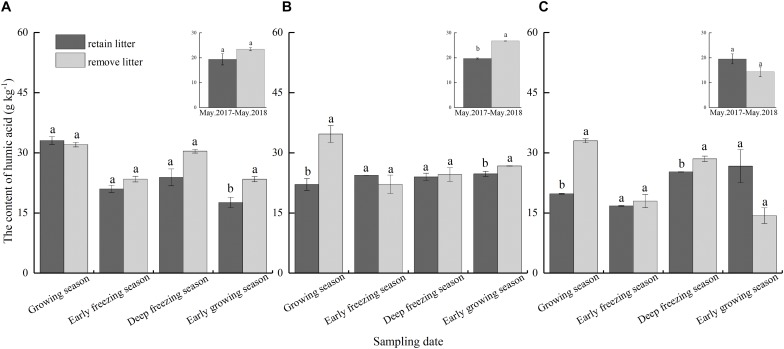
Content of soil humic acid as affected by litter removal at different periods in the **(A)** coniferous forest, **(B)** mixed forest, and **(C)** broadleaved forest. The histograms show the averaged values over 1 year. The presented content values are the means of 3 observations; the error bars represent standard errors. Lowercase letters represent significant differences in the contents of total extracted humus between the different litterfall treatments.

Similar to the total extracted humus, litterfall significantly affected the HA content in the soil (*F* = 25.25, *P* < 0.01, Table 2), and there were significant differences between different forests and seasons (Table 2). The content of HA in the retained litterfall soil was considerably decreased by 26.3% in the mixed forest but not in the coniferous and broadleaved forests after the 1-year incubation. Litter input significantly reduced by 24.8% soil HA content in the coniferous forest during the early growing season (Figure 4A). Litter input also significantly reduced the soil HA content in the mixed forest during the growing season and the early growing season, with a reduction of 36.3 and 7.4%, respectively (Figure 4B). Moreover, litterfall significantly decreased the soil HA content of the broadleaved forest in the growing season and deep freezing season, resulting in a 40.1 and 11.4% reduction, respectively (Figure 4C).

### Fulvic Acid

**FIGURE 5:**
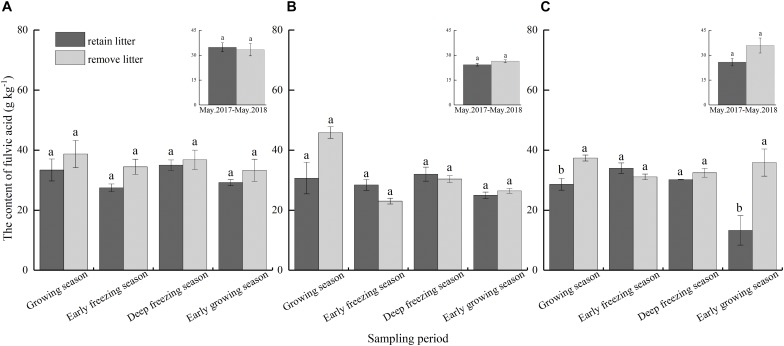
Content of soil fulvic acid as affected by litter removal at different periods in the **(A)** coniferous forest, **(B)** mixed forest, and **(C)** broadleaved forest. The histograms show the averaged values over 1 year. The presented content values are the means of 3 observations; the error bars represent standard errors. Lowercase letters represent significant differences in the contents of total extracted humus between the different litterfall treatments.

The results showed that litterfall significantly affected the content of soil FA (*F* = 11.36, *P* < 0.05, Table 2), and there were significant differences between different seasons (Table 2). The continuous input of litter for 1 year had no significant effect on the content of FA in these three forests (Figure 5). In addition, seasonal litterfall input significantly reduced the content of soil FA in the broadleaved forest only during the early growing season and the growing season (Figure 5C), with a reduction of 63.0 and 23.4%, respectively; there were no significant effects in the other periods and forests.

### HA/FA Ratios

**FIGURE 6:**
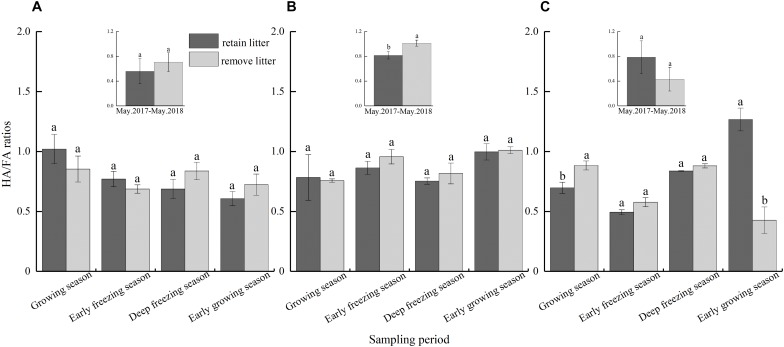
The HA/FA ratios as affected by litter removal at different periods in the **(A)** coniferous forest, **(B)** mixed forest, and **(C)** broadleaved forest. The histograms show the averaged values over 1 year. The presented content values are the means of 3 observations; the error bars represent standard errors. Lowercase letters represent significant differences in the contents of total extracted humus between the different litterfall treatments.

The effects of continuous litterfall input for 1 year on the HA/FA ratios of the three types of forest differed. The HA/FA ratio of the mixed forest (Figure 6B) was reduced significantly, although non-significant effects were detected in the coniferous and broadleaved forests (Figures 6A,C). Seasonal litterfall significantly reduced the HA/FA ratio of the broadleaved forest in the growing season and increased the ratio during the early growing season. Litterfall did not have statistically significant effect on the soil HA/FA ratios in the coniferous and mixed forests in all periods, and the values were always less than 1 (Figures 6A,B).

### Correlations Between the Extracted Soil Humic Substances and the Environment

The analysis showed that litterfall changed the relationships between the soil humic substances and the selected environmental factors. There was no significant correlation between the soil humic substances and the environmental factors in the litter retained plots in all three forests (Table 3). However, the content of humic substances in the removed litterfall soil was significantly positively correlated with the relative humidity, daily mean temperature, positive accumulated temperature and negative accumulated temperature but was negatively correlated with the number of freeze-thaw cycles in all studied forest types (Table 3).

**TABLE 3:** Correlation analyses between the total extracted humus, HA, FA, HA/FA ratios and environmental factors with different litterfall treatments.

	Soil retained litter				Soil removed litter			
	Total extracted humus	HA	FA	HA/FA	Total extracted humus	HA	FA	HA/FA
Relative humidity	0.033	0.108	–0.041	0.129	0.537**	0.431**	0.468**	0.021
Daily mean temperature	–0.067	0.199	–0.212	0.297	0.523**	0.405*	0.470**	0.018
Positive accumulated temperature	0.188	0.217	0.047	0.137	0.715**	0.624**	0.577**	0.112
Negative accumulated temperature	–0.178	0.184	–0.32	0.356*	0.392*	0.272	0.380*	–0.022
Number of freeze-thaw cycles	0.058	–0.216	0.214	–0.308	−0.531**	−0.416*	−0.472**	–0.028

*P < 0.05, **P < 0.01. *n* = 36.

## Discussion

Litterfall transfers approximately one third of the global annual C uptake (approximately 18 Pg⋅C⋅year^–1^) to soil surface (Grace, 2004; Malhi et al., 2011), although the amount of this C that is sequestered in soils is not fully understood. Humic substances in the soil are in a dynamic process of continuous decomposition and synthesis (Stevenson, 1994), and the amount depends on the relative amounts of the formation and degradation of humic substances (Dou et al., 2007). Our results partly supported our hypothesis that retained litter may decrease soil humic substances content, but this effect may be regulated by litter types and litter production in different periods. Our results show that litterfall promoted a reduction in soil humic substances in the mixed forest but had insignificant effect in the coniferous forest and broadleaved forest. In addition, seasonal litterfall input significantly promoted a reduction in soil total extracted humus in the three forests during the growing season. This finding suggests that the effects of litterfall on soil humic substances were related to the litterfall types. Moreover, a greater amount of seasonal litterfall corresponded to a greater reduction in soil total extracted humus.

### Total Extracted Humus

Over 1 year continuous litterfall input, we found that the soil total extracted humus in the mixed forest decreased significantly, indicating that the degradation rate was higher than the synthesis rate in the 1-year incubation. However, litterfall did not have a significant effect on soil extracted humus in coniferous or broadleaved forest. The formation and degradation of humic substances mainly depend on the action of microorganisms (Cotrufo et al., 2013). Litterfall input increases the available carbon source of soil microorganisms (Nadelhoffer et al., 2004), which provides important energy for decomposer activities and stimulates the decomposition or mineralization of soil humic substances that originally existed in the soil (Kuzyakov, 2010; Cotrufo et al., 2013). Moreover, the decomposition rate of mixed forest litterfall has been demonstrated to be faster than that of single litter type (Briones and Ineson, 1996; Hooper, 1998; Chapman and Koch, 2007), and it can enrich the microbial community structure and provide more nutrients for soil microbes to promote their activity (Nadelhoffer et al., 2004), thereby promoting the reduction of soil humic substances. The input of litterfall also provides raw materials for the synthesis of humic substances and promotes their synthesis. Single litter type, such as needle leaves in the coniferous forest and broad leaves in broadleaved forest, decompose slowly and accumulate more refractory substances (Ponge, 2013), which can promote the synthesis of humus and balance the decomposition of humic substances to a certain extent (Gregorich et al., 1996). These results indicate that the effect of litterfall on the soil humic substances is closely related to the litterfall types (Wei et al., 2018).

In the present study, the total extracted humus in the three forests was decreased because of litterfall during the early growing season and the growing season. During the growing season, the greater the amount of litterfall was, the greater the reduction amount of soil total extracted humus, suggesting that the degradation of humus was affected by the amount of litterfall. However, the amount of litterfall during the early growing season occurred in the order of coniferous forest > mixed forest > broadleaved forest (Figure 2), but the amount of reduction occurred in the order of broadleaved forest > coniferous forest > mixed forest (Figure 3). This result may be because the needle litter contained less simple and soluble substrates (Berg, 2000) and was high in C/N, cellulose and lignin (Dai et al., 2001), which have difficulty decomposing and form humus easily (Taylor, 1989; Cotrufo and Ineson, 1995).

### Humic Acid, Fulvic Acid and HA/FA Ratios

Soil HA and FA are the main components of soil humic substances, but these two acids have different stability and formation processes. We found that soil HA content and HA/FA ratio significantly decreased in mixed forests after 1 year of continuous litterfall input, but FA content was not affected by litter input. Moreover, the reduction in the soil HA content of mixed forest was more than total extracted humus, which may be due to the conversion of HA and FA (Stevenson, 1994) or the priority synthesis of FA in the synthesis of humus (Dou et al., 2007; Ni et al., 2014, 2016). The effect of seasonal litterfall input on the soil HA was similar to that of the total extracted humus (Figure 4). However, seasonal litterfall significantly reduced the content of HA only in the broadleaved forest during the growing season and early growing season and had no significant effect in the coniferous and mixed forests (Figure 5), which may be related to the litterfall quality and components. The litterfall of the coniferous and mixed forests contained needle litter (Figures 2A,B), which contained terpenoids and phenolic substances (Wu et al., 1993; Shen and Bartha, 1997), and was more likely to cause acidic environments during decomposition (Yang et al., 2007). Acidic environments are more conducive to the synthesis of FA (Dou et al., 2010), and therefore the reductions in soil FA in the coniferous and mixed forests were non-significant. The HA/FA ratios of these forests were less than 1 (Figure 6), suggesting that the synthesis rate of FA was always higher than that of HA. Litterfall had no significant effect on the HA/FA ratios in the coniferous and mixed forests among the different periods, while litterfall significantly decreased the ratio in the broadleaved forest in the early growing season. This result might be due to the fact that litterfall promoted the conversion between HA and FA (Stevenson, 1994). Furthermore, litterfall had no significant effect on the content of soil humic substances in winter. This finding may be because low temperatures and soil freezing inhibited soil microbial activity (Bokhorst et al., 2013) and hindered the physiological metabolism of the microorganisms involved in the formation and degradation of humic substances (Cotrufo et al., 2013).

Litterfall promoted a reduction in soil humic substances mainly in the growing season. Moreover, seasonal litterfall input had a more significant effect on the soil humic substances than continuous litterfall input. The seasonal litterfall input mainly relied on the rapid input of available carbon sources to promote soil microbial activity (Kuzyakov, 2010). After the rapid consumption of soluble carbon sources, continuous litterfall input was mainly dependent on the decomposition of components that are not easily decomposed, such as cellulose and lignin, to provide nutrients to the soil (Kumada et al., 1967). Therefore, we suspected that the input of easily decomposable substances mainly promoted the decomposition of soil humic substances while the input of substances that had difficultly decomposing mainly promoted the synthesis of humus (Fontaine et al., 2003). However, the decomposition of substances that have difficultly decomposing and the synthesis of humic substances are slow processes; therefore, they were not shown in this study.

### Correlations Between the Extracted Soil Humic Substances and the Environment

A significant difference was observed between the content of humic substances in the removed litter soil and the environment, although few significant correlations were observed in the retained litter soil (Table 3). This result indicates that litterfall protected or buffered the soil humic substances, making them less susceptible to climatic factors. Furthermore, the content of humic substances in the removed-litter soil might be regulated by biological factors, such as microbial activity. Furthermore, the content of humic substances was significantly positively correlated with the relative humidity, daily mean temperature, positive accumulated temperature and negative accumulated temperature but negatively correlated with the number of freeze-thaw cycles (Table 3). This finding indicates that an increase in temperature affected the content of soil humic substances, and the frequent freeze-thaw cycles in winter may inhibit the formation of humus and even destroy newly formed humus to cause degradation (Dou, 2010). Therefore, under the background of global warming, litterfall can weaken the effects of frequent freeze-thaw cycles (Sahin et al., 2008) and temperature increases (Easterling et al., 1997) on the content of soil humic substances and play a protective role for soil humus.

## Conclusion

Litterfall significantly decreased the soil humic substances and HA content of three forests during the growing season but showed insignificant effects in freezing season, implying that a longer growing season and shorter winter caused by global warming may promote the degradation of soil humic substances and the potential loss of soil organic matter. We also found that the relationship between litterfall and soil humus is related to the amount of litterfall during 1-year incubation, displaying a greater increase in the amount of litterfall corresponding to a greater decrease in the content of soil humic substances. Furthermore, a lack of litterfall could increase the sensitivity of soil humic substances to environmental factors. These results showed that, the less litterfall input in a short term, the more conducive to the accumulation of soil humic substances, but the effect of long-term litterfall input on soil humic substances content required further research. This study provided some basic evidence for understanding plant-soil interactions in the subalpine forests.

## Data Availability Statement

The raw data supporting the conclusions of this manuscript will be made available by the authors, without undue reservation, to any qualified researcher.

## Author Contributions

LZ and FW conceived the study, designed the experiments, and supervised the whole study. XW, YY, YS, ZC, and YD performed the experiments. XW, LZ, and FW wrote the manuscript.

## Conflict of Interest

The authors declare that the research was conducted in the absence of any commercial or financial relationships that could be construed as a potential conflict of interest.
